# Deep learning‐based classification of organs at risk and delineation guideline in pelvic cancer radiation therapy

**DOI:** 10.1002/acm2.14022

**Published:** 2023-05-12

**Authors:** Michael Lempart, Jonas Scherman, Martin P. Nilsson, Christian Jamtheim Gustafsson

**Affiliations:** ^1^ Radiation Physics, Department of Hematology Oncology, and Radiation Physics Skåne University Hospital Lund Sweden; ^2^ Department of Translational Medicine Medical Radiation Physics Lund University Malmö Sweden; ^3^ Department of Hematology Oncology, and Radiation Physics Skåne University Hospital Lund Sweden

**Keywords:** deep learning, guideline, organs at risk, radiation therapy, structure classification

## Abstract

Deep learning (DL) models for radiation therapy (RT) image segmentation require accurately annotated training data. Multiple organ delineation guidelines exist; however, information on the used guideline is not provided with the delineation. Extraction of training data with coherent guidelines can therefore be challenging. We present a supervised classification method for pelvis structure delineations where bowel cavity, femoral heads, bladder, and rectum data, with two guidelines, were classified. The impact on DL‐based segmentation quality using mixed guideline training data was also demonstrated. Bowel cavity was manually delineated on CT images for anal cancer patients (*n* = 170) according to guidelines Devisetty and RTOG. The DL segmentation quality from using training data with coherent or mixed guidelines was investigated. A supervised 3D squeeze‐and‐excite SENet‐154 model was trained to classify two bowel cavity delineation guidelines. In addition, a pelvis CT dataset with manual delineations from prostate cancer patients (*n* = 1854) was used where data with an alternative guideline for femoral heads, rectum, and bladder were generated using commercial software. The model was evaluated on internal (*n* = 200) and external test data (*n* = 99). By using mixed, compared to coherent, delineation guideline training data mean DICE score decreased 3% units, mean Hausdorff distance (95%) increased 5 mm and mean surface distance (MSD) increased 1 mm. The classification of bowel cavity test data achieved 99.8% unweighted classification accuracy, 99.9% macro average precision, 97.2% macro average recall, and 98.5% macro average F1. Corresponding metrics for the pelvis internal test data were all 99% or above and for the external pelvis test data they were 96.3%, 96.6%, 93.3%, and 94.6%. Impaired segmentation performance was observed for training data with mixed guidelines. The DL delineation classification models achieved excellent results on internal and external test data. This can facilitate automated guideline‐specific data extraction while avoiding the need for consistent and correct structure labels.

## 1 INTRODUCTION

The use of machine learning enables automation and optimization of the clinical radiation oncology workflow. Deep learning (DL), a subfield of machine learning, is well suited to be used for radiation oncology due to the large amount of available image data. Several reviews on the use of DL and its applications in radiation therapy (RT) have been written.^1^, ^2^, ^3^, ^4^, ^5^, ^6^, ^7^, ^8^, ^9^ Deep learning‐based models can extract and learn features from images and the most common applications within radiation oncology are the generation of synthetic computed tomography (CT) volumes, automatic treatment planning, and image segmentation.^9^, ^10^ In this paper we will refer to the manual definition of organ boundaries as organ delineation and the automatic process as semantic segmentation. The use of DL for semantic segmentation has matured into several commercially available products for RT organs at risk (OAR) segmentation, achieving similar segmentation quality with decreased human user variability,^3^, ^11^ compared to manual delineations. This automation has also shown to decrease the overall time needed for OAR segmentation,^12^, ^13^ and time savings up to 70% percent, including manual corrections, have been demonstrated.^14^ To reach acceptable DL model generalization training data must contain a wide range of diverse subject data to learn from, often meaning the dataset must contain several thousands of training images with a representative data distribution.^15^ In RT, instructions on organ delineation are called delineation guidelines and several different guidelines for the delineation of the same organ exists and are used all over the world. The training dataset should be cleaned and carefully inspected prior model training. Extraction of data over long timeframes might also be subject to changing delineation guidelines. Further, data extraction from multiple sites can be associated with difference in delineation guidelines. For optimal DL segmentation quality it is crucial that the data contains low noise, is of high quality,^16^, ^17^ and that delineation guidelines are coherent, as demonstrated in this paper.

Each OAR and target volume has a structure name in form of a text label attached to it, where the label should be standardized. Such naming standardization would facilitate data extraction to build large training datasets^18^ and several methods utilizing this have been published.^19^, ^20^, ^21^, ^22^ A well‐used naming schema has been presented by the American Association of Physicists in Medicine (AAPM) Task Group 263 (TG‐263).^23^ In Sweden the University Hospitals aim to follow the naming standard defined in Santanam et al.^24^ and ICRU,^25^ see further details.^20^, ^26^

It is however important to highlight that text‐based methods only accounts for differences in the labels and assume that the RT structure delineation image data is representative and correct.^27^, ^28^ Further, labels do not contain information on the used delineation guidelines. For large data collection, with the purpose of training DL models, the lack of information in the label can constitute a problem that needs attention. Cleaning, renaming, and quality control of the data are required when creating high‐quality datasets for machine learning.^17^, ^29^ This can be heavily time consuming^19^ and can reduce the available research time.^30^ Automation of this process would therefore be of high value motivating the development of image‐based machine learning methods for RT structure classification. Such methods for lung and heart^27^, ^28^ have been demonstrated and DL image‐based classification with convolutional neural networks (CNNs) have been published for head and neck^31^, ^32^, ^33^ and prostate.^32^, ^33^

In previous work, the delineation structure contents were analyzed from a geometrical point of view using a 2D DL CNN classification network.^18^ A classification accuracy of 99% was achieved on internal test datasets, consisting of CT and magnetic resonance imaging (MRI) images where structure delineations could be relabeled to any naming standard. The developed model showed improved class diversity and usefulness for uncleaned clinical data, compared to previous publications and models.^22^, ^28^ The results also demonstrated the importance of specific delineation guideline knowledge (despite coherent labeling), as inferior results were seen on external test data with difference in delineation guidelines.

In this work, we build upon an existing method,^18^ focusing on supervised classification and differentiation between delineation guidelines for pelvis RT structures. We present a method for pelvis CT image data classification where data of interest can automatically be classified and extracted. The used classification model has been trained in 3D, as opposed to previous work which utilized 2D images and has a dedicated classification class for structures that are not of interest, thereby decreasing false positives. We argue that precision should be prioritized in the classification task to select data with coherent delineation guidelines. We, therefore, used a custom precision metric to control model training. We further demonstrate differentiation between two types of bowel cavity, femoral heads, bladder, and rectum delineation guidelines.

The aim of this work was to (1) demonstrate impaired performance for DL semantic segmentation when training data contains non‐coherent and multiple delineation guidelines and (2) develop, evaluate, and verify deep learning‐based classification models to differentiate pelvic OAR structures with major and minor differences in delineation guidelines.

## 2 METHODS

### 2.1 Demonstration of impaired segmentation performance using mixed training data

In Lempart et al.^14^ a deep‐learning model, referred to as Pelvic U‑Net, for multi‐label semantic segmentation of pelvic OAR 3D structures such as total bone marrow, rectum, bladder, and bowel structures was developed, and demonstrated clinically acceptable segmentations with average time savings of 70%. The same model was used in this work to demonstrate the effect on segmentation performance when the model was trained with data containing a mixture of two different delineation guidelines.

**FIGURE 1:**
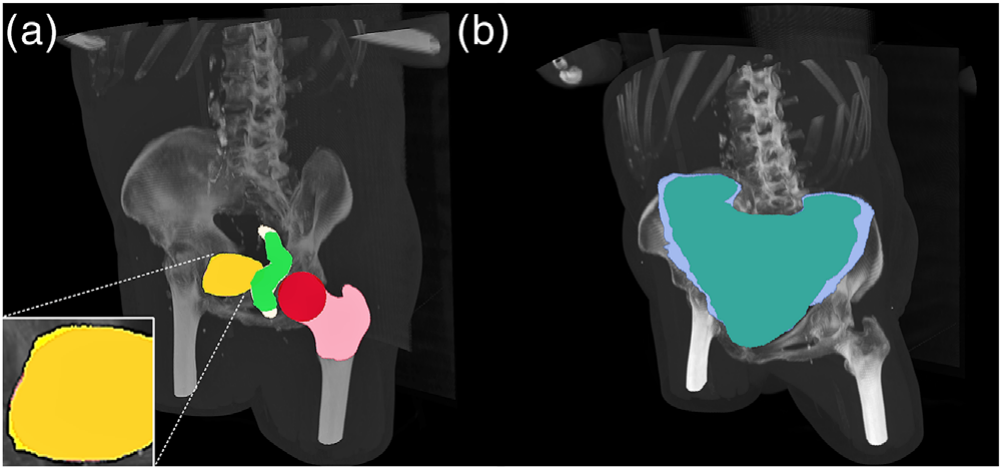
Organs at risk (OAR) of interest visualized for a subject example case, showing the difference in delineation guidelines for the pelvis dataset (a) and the bowel dataset (b). In (a), the lighter, larger, yellow color represents the clinical bladder structure where the bladder wall is included, see zoomed in area. The rectum structure from the AI semantic segmentation software (shown in white) has a larger inferior and superior coverage compared to the clinical rectum structure in green. The femoral head structure from the AI segmentation software in pink includes, apart from the femoral head itself, a larger part of the femoral bone compared to the clinical spherical femoral head in red. In (b), the RTOG delineation guideline in lighter blue is larger than the Devisetty delineation guideline in Bluegreen.

The dataset used in the demonstration consisted of a total of 170 patients where 12 patients had hip prothesis and were excluded, hence a total of 158 patients were used to generate training, validation, and test datasets. All patients had squamous cell carcinoma of the anus (anal cancer) and were treated with RT at Skåne University Hospital, Lund, Sweden, during the period August 2009 to December 2017. Original study ethics was approved by the Regional Ethics Board of Lund, Sweden (EPN Lund, Dnr 2013/742). The bowel cavity of all 158 patients in the dataset was manually delineated on a CT volume according to the Devisetty^34^ delineation guideline, see Lempart et al.^14^ for details (Figure 1b). In addition, the bowel cavity was manually delineated by the same oncologist according to the RTOG^35^ delineation guideline. This dataset will be referred to as the bowel dataset and several different CT scanners were used to acquire the data.

Using the bowel dataset, three different Pelvic U‑Net models were trained for segmentation of the bowel cavity structure. Model training was performed using 5‐fold cross validation (80%/20% split), as in Lempart et al.^14^ Training and validation data for all three models consisted of a total of 143 patients without hip prothesis where training and validation data for the first model only contained patients with manual bowel cavity delineations according to Devisetty,^34^ further referred to as ModelDev. The second model contained manual delineations according to RTOG,^35^ further referred to as ModelRTOG. The third model was trained with data consisting of a 50%/50% mixture of the Devisetty and RTOG delineation guidelines, further referred to as ModelMix.

ModelDev and ModelRTOG were evaluated on an independent test dataset (also referred to as a holdout dataset in the literature) consisting of 15 random patients, with no patient overlap compared to training and validation data, using the same delineation guideline as the corresponding training data. ModelMix was evaluated twice on the same test dataset with 15 patients, one time with Devisetty and one time with RTOG delineation guideline test data. Using an average model ensemble based on the models discovered via the cross‐validation folds, model inference was carried out on all test datasets. The DL generated semantic segmentations were truncated above the ground truth segmentations before geometric evaluation using the Dice similarity coefficient (DSC), the 95th percentile of the Hausdorff distance (HD95), and the symmetric mean surface distance (MSD).^14^

A statistical comparison of the geometric metrics originating from ModelDev, evaluated on the Devisetty test dataset, and ModelMix, evaluated on the Devisetty test dataset, was performed using a non‐parametric, two‐sided Wilcoxon signed rank test, with a significance level of 0.05. Similarly, the same statistical test was applied to geometric metrics originating from ModelRTOG, evaluated on the RTOG test dataset, and ModelMix, evaluated on the RTOG test dataset.

### 2.2 Development of deep learning‐based classification with delineation guideline differentiation

#### 2.2.1 Dataset generation and analysis—bowel dataset

The bowel dataset of 170 patients, including the 12 patients with hip implants, was also used for RT 3D structure label classification. A training and validation dataset with a total of 145 patients were randomly selected from the complete bowel dataset, each patient containing the two bowel cavity delineation guidelines Devisetty and RTOG as Digital Imaging and Communications in Medicine (DICOM) RT structures. In total, the 145 patients consisted of 5766 different 3D structures. An independent test dataset of 25 patients was randomly selected from the complete bowel dataset, containing a total of 975 3D structures, where each patient contained the two bowel cavity structure delineations.

#### 2.2.2 Pelvis dataset

An additional dataset with 2054 patients, originally used in Jamtheim Gustafsson et al.,^18^ here referred to as the pelvis dataset, contained patients who received external beam RT between June 2016 and August 2020 using volumetric modulated arc therapy (VMAT). 1854 patients were chosen for training and validation and 200 as an independent internal test dataset. All delineations originated from the same clinic and were manually created by several radiation oncologists and treatment planning staff using an internal clinical delineation guideline.

To generate 3D semantic segmentations with an alternative guideline^35^ for the pelvis dataset, all 2054 patient CT volumes were parsed through the RaySearch male pelvic CT model version 1.0.0 (RaySearch Laboratories, Stockholm, Sweden), an AI‐based segmentation software, using a custom Python script. Compared to the clinical guidelines, the RaySearch male pelvic CT model was trained to segment the rectum structure with larger coverage in the inferior and superior direction and the femoral heads to include a part of the femoral bone. In addition, the bladder wall was excluded from the bladder in the AI‐based segmentations. For further details and guideline differences, see Figure 1. After automatic semantic 3D segmentation, a DICOM RT structure set containing segmentations for femoral heads (Femur_Head_L_AI1 and Femur_Head_R_AI1), rectum (Anorectum_AI1), and bladder (Bladder_AI1) was returned for each patient. If hip implants were present in the patient, the segmentation software still attempted to generate a structure for the femoral head. Nevertheless, the RaySearch male pelvic CT model was not trained for such implants. The total size of the pelvis dataset could thereby be increased, containing two different delineation guidelines. After this action, the training dataset contained to 40 698 3D structures and the test set contained 4574 3D structures.

#### 2.2.3 External test dataset

In addition to the internal test dataset, an external dataset with 99 prostate cancer patients who received external beam RT between 2011 and 2017, from the RT clinic at Umeå University Hospital, Umeå, Sweden was available through a common collaboration, see Jamtheim Gustafsson et al.^18^ for details. Patients were delineated using internal clinical guidelines and two patients had hip prothesis. 3D structures with an additional delineation guideline for femoral heads, rectum, and bladder were created for this dataset with the RaySearch male pelvic CT model as described above and the total dataset contained 1798 3D structures. This dataset is referred to as the external test dataset.

### 2.3 Data preprocessing

**FIGURE 2:**
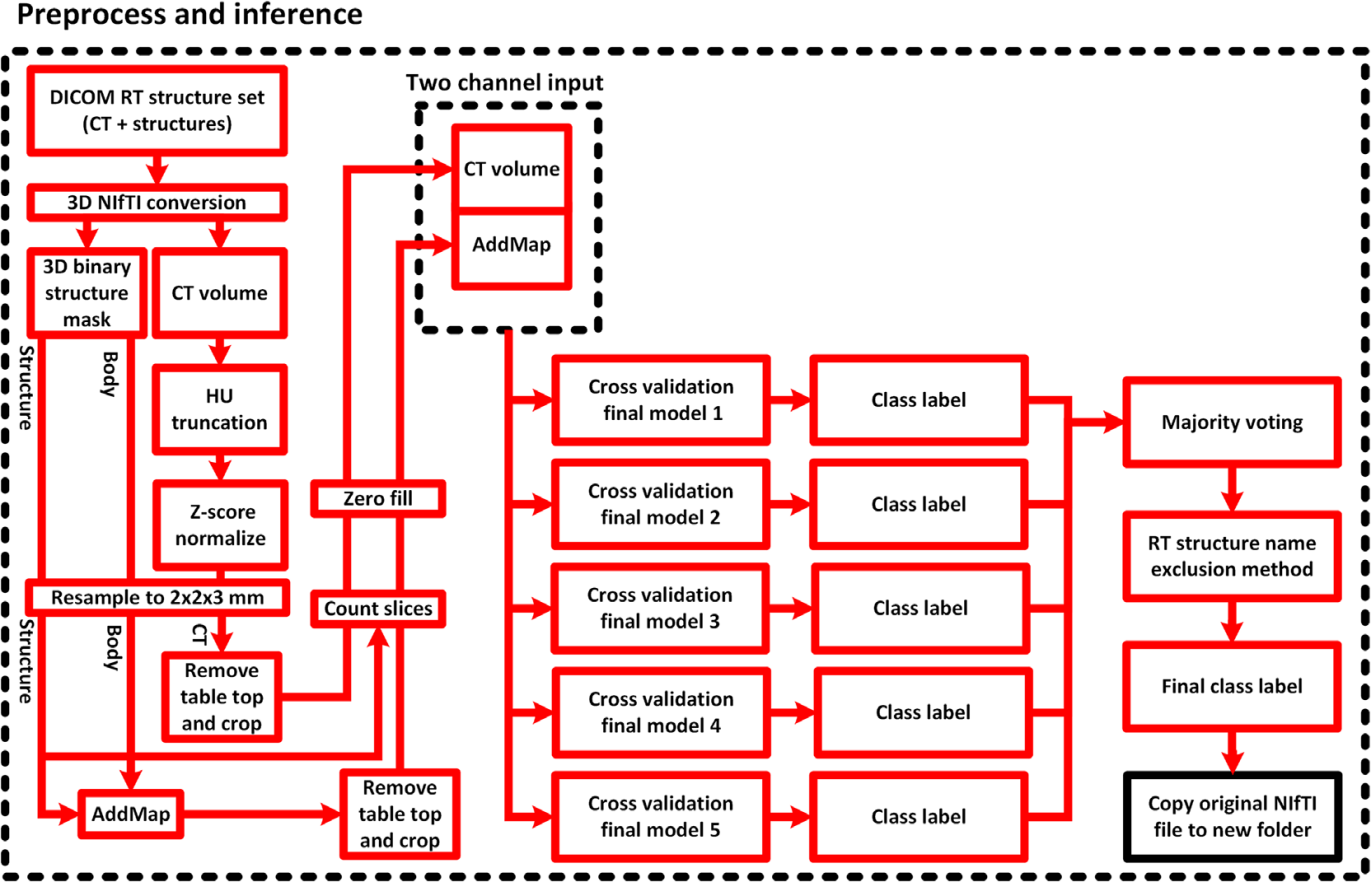
A block diagram of the method workflow describing preprocess and model inference. The preprocess compartment contains the necessary steps for extracting and preprocessing the DICOM radiation therapy (RT) structure data to the desired format. CT volume and AddMap data are provided to the cross‐validation models, following a majority vote among the models. The final class label can depend on if the structure label is within the structure name exclusion list. After the final class label has been determined the original structure data is copied to a new folder.

The methods for data preprocessing and model training (where applicable) were the same for the bowel dataset, pelvis dataset and the external dataset if not mentioned otherwise below. Code for preprocessing of data, model training, and model inference is available at https://github.com/jamtheim/DicomRTGuidelineClassifierPublic. A block diagram of the data preprocessing and inference pipeline is presented in Figure 2.

The transversal 3D DICOM CT volume data together with the 3D RT structures were anonymized and converted to 3D NIfTI^36^ using the Python package dcmrtstruct2nii (v.1.0.19),^37^ where each RT structure was represented by a 3D binary mask. All NIfTI files for each patient were collected in a separate folder. The body structure could be identified by the name “BODY” or “External,” contained in the DICOM files. The CT volumes and structures were read to Python Numpy format from the NIfTI file using SimpleITK.^38^

Each patient was z‐score normalized where the mean value and standard deviation in the normalization were calculated from image volume voxels with Hounsfield units (HUs) larger than −1000 (air). Normalized CT data and body structures were resampled to 2 × 2 mm in plane spatial resolution and 3 mm slice thickness using bi‐quadratic and nearest neighbor interpolation, respectively. The tabletop was removed from the CT data using the available body structure and CT data was thereafter cropped to the bounding box.

Each non‐empty RT structure NIfTI file was read and resampled to the same resolution as the CT, using nearest neighbor interpolation. Anything outside of the body contour, such as the tabletop, was removed by volume masking. A new 3D image volume was created by adding the resampled body binary mask and each resampled structure binary mask, and dividing the sum by two. This yielded a maximum value of one for the body and structure overlap, 0.5 for the body structure and zero for the background. This is referred to as an AddMap^18^ and provided the neural network with spatial 3D information on both the structure geometry and its spatial surrounding with respect to the location of the 3D body structure. This additional geometry information aided the neural network's ability to learn the classification features, see Figure 2 in Jamtheim Gustafsson et al.^18^ The structure together with the AddMap was cropped using a bounding box, calculated using the resampled body structures. The number of 2D slices in the 3D structure containing pixel values larger than 0 was counted, and if larger than 96 (96 × 3 mm = 288 mm inferior/superior), the structure was ignored, that is, support for large 3D structures was limited, but the tabletop could in this way be ignored. For each 3D structure, the 2D center slice index, and a slice range of ±96/2 slices was calculated, where the center slice index was positioned in the middle of the range. CT and AddMap were then truncated to this range in the inferior/superior direction, providing unique CT and AddMap data for each structure. CT and AddMap were symmetrically zero‐filled (when needed) to the image size 184 × 280 × 96 for the bowel dataset and to 200 × 328 × 96 for the pelvis dataset and external dataset. Difference in resulting in‐plane image size between these was due to difference in physical patient size between the two datasets. The final CT and AddMap data were then saved to a 4D Numpy array where CT and AddMap were saved in separate 3D image channels, see Figure 2.

### 2.4 Model input, configuration, and training

As the task was defined as a classification problem, a 3D ResNeXt CNN backbone, built upon ResNet, was selected due to its previous success and use in both general^39^, ^40^ and medical image classification.^18^ To adaptively recalibrate channel‐wise feature responses and to model channel interdependencies, the ResNeXt backbone was combined with Squeeze and excite blocks^41^ into a 154 layer model referred to as SENet‐154.^41^ All model training and inference was performed using PyTorch‐lightning 1.6.0^42^ and the SENet‐154 model was loaded from the MONAI framework (v.0.8.1).^43^ The CNN was trained in a supervised manner and model weights were initialized with He initialization as prescribed by Pytorch‐lightning default.^44^ The input to the model consisted of two image channels, one with the CT data and one with the AddMap, preprocessed according to section 2.3 *Data preprocessing*.

One multiclass SENet‐154 classification model was trained separately for the bowel dataset and another one was trained separately for the pelvis dataset. For the bowel dataset, three classification labels were defined as (1) bowel cavity with Devisetty delineation guideline, bowel cavity with RTOG delineation guideline and (2) an “Other” class for all other structures. For the pelvis dataset, a total of nine classification labels were defined as “Bladder,” “Bladder_AI1,” “FemoralHead_L,” “Femur_Head_L_AI1,” “FemoralHead_R,” “Femur_Head_R_AI1,” “Rectum,” “Anorectum_AI1,” and “Other” where the suffix “_AI1” defined structures generated from the RaySearch male pelvic CT model. The “Other” class representation was used to facilitate extraction of data with lower false positive rate as this was identified as a problem in Jamtheim Gustafsson et al.^18^

For model training, a weighted categorical cross entropy loss was used where the class weights were calculated as a balanced distribution using the Scikit‐learn Python package,^45^ that is, the inverse of the label frequency distribution was used as weights. The class label imbalances originated from differences in number of available structures for each class. A probability value for each of the defined classes was derived at the networks output, where the largest probability value determined the final output class. The adaptive moment estimation (Adam) was chosen as an optimizer, with a learning rate of 0.0001.^46^ The learning rate was monitored for each epoch and multiplied with 0.5 if no change in validation loss was detected within 15 epochs. Mixed precision training was used to lower GPU memory consumption.

As classification precision was prioritized in the training, a custom precision metric was defined for the validation data as the sum of the model precision calculated for each class, except the “Other” class, see Equation (1).

(1) $$\mathrm{CP}=\sum_{\begin{array}{c} i=1\\ i\neq j \end{array}}^{n}\left(P_{i}\right)$$

where CP is custom precision, *P_i_* is the class precision for class *i*, *n* is number of classes and *j* is the index for the “Other” class.

Early stopping was utilized by monitoring the custom precision metric where the training was stopped if no improvement in the custom precision metric was detected for 60 epochs for the bowel dataset and 20 epochs for the pelvis dataset. The number of epochs given was empirically determined.

To increase the amount of training data and to avoid overfitting, on‐the‐fly 2D in‐plane augmentation for image and ground truth was performed using left‐right flipping, rotation from −5° to 5° with nearest neighbor interpolation and translation of −10 to 10 pixels in each dimension. For training on the bowel dataset, each augmentation had a probability of 0.5, one GPU was used, and the batch size was set to 6. For training on the pelvis dataset, augmentation consisted of rotation and translation, each with a probability of 0.4, trained on two GPUs in distributed data parallel mode with a batch size of 5 for each GPU.

The training was performed using 5‐fold cross validation and the classification performance metrics, precision, recall, sensitivity, and F1 score were calculated globally and per class together with global unweighted average (macro avg) and weighted average (weighted avg) performance measures. See Supplementary material for metric definitions. The training was monitored using Tensorboard^47^ and the best model, with respect to the highest validation custom precision metric, was saved for each cross validation.

From the large patient cohort in the pelvis dataset, we also investigated the CNN classification performance as a function of number of subjects in the training data. This was of interest due to the minor delineation guideline differences existing between the clinical and AI segmented bladder structure (Figure 1). The experiment was performed by training the model only once, using the first cross validation fold with 100, 200, 300, 400, 500, 600, 700, 800, and 1840 subjects and assessing the accuracy on validation data (20%).

Dataset generation, calculations, and model training were performed with an AMD Ryzen Threadripper 3970 × 64 thread CPU, 256 GB RAM combined with one or two NVIDIA 3090 RTX GPUs with NVLink, each with 24 GB RAM, running Ubuntu 20.04.4 LTS with CUDA v.11.1.105 and NVIDIA driver version 510.54. The source code is available on GitHub at https://github.com/jamtheim/DicomRTGuidelineClassifierPublic.

### 2.5 Model inference

The model inference was designed to be compatible with both DICOM and NIfTI data formats, where DICOM data was automatically converted to NIfTI format. The same preprocessing was applied to the test data as for the training data, with the addition of image downscaling if the image volume after preprocessing still had a larger in‐plane size than desired, see section 2.3 *Data preprocessing*. Inference was performed with mixed precision using one GPU with batch size 1 using the best model from each fold in the 5‐fold cross validation. Each RT structure in each patient was thereby classified by each model into one of the class labels. A majority voting between all the five cross validation models was performed, classification metrics was calculated, and all results were written to an Excel file. In addition to this, the structures were copied to separate output folders, depending on determined majority vote class.

The inference process described above was also repeated in a separate experiment where the final class label for each structure after majority voting was also dependent on the RT structure name, method referred to as “RT structure name exclusion.” The inference procedure was defined like this: After the class label was determined from the CNN, as described above, a text‐based control check was performed to determine if any of the predetermined exclusion keywords were included in the RT structure name (lowercase comparison). If this was true, the class label was set to “Other.” This was performed to increase the classification precision. The exclusion keywords for the bowel dataset were “tuning,” “HELP,” “X_,” “Y_,” “opt_,” “Dose,” “Match,” “Artefakter,” “Artifakter,” “artefakt,” “GTV,” “CTV,” “PTV,” “ring,” “bowelbag,” “abdomen,” “tarm,” “buk,” and “peritoneum.” For the pelvis dataset the exclusion keywords were “tuning,” “HELP,” “X_,” “Y_,” “Z_,” “opt,” “Dose,” “Match,” “Artefakter,” “Artifakter,” “artefakt,” “GTV,” “CTV,” “PTV,” “ring,” and “AnalCanal.” These keywords were chosen without prior knowledge on the full structure contents of the test datasets.

## 3 RESULTS

### 3.1 Demonstration of impaired segmentation performance using mixed training data

ModelDev, trained on the bowel cavity dataset delineated according to Devisetty (Table S1), and ModelRTOG, trained on the bowel cavity dataset delineated according to the RTOG delineation guidelines (Table S2), were evaluated on an independent test dataset with 15 patients using the respective delineation guideline. The results showed a mean DSC (±1 STD) of 0.95 ± 0.01 and 0.94 ± 0.01, respectively. Corresponding values for HD95 (±1 STD) were 4.49 ± 1.72 mm and 5.22 ± 1.38 mm. For the MSD (±1 STD), values of 1.04 ± 0.31 mm and 1.17 ± 0.19 mm were found. ModelMix, applied to Devisetty test data (Table S3), showed a mean DCS of 0.92 ± 0.03 and applied to RTOG test data (Table S4) 0.91 ± 0.03 (both lower and inferior). Corresponding values for HD95 were 8.94 ± 4.89 mm and 10.16 ± 4.72 mm, respectively (both larger and inferior). Corresponding values for MSD were 1.97 ± 0.86 mm and 2.12 ± 0.8 mm (both larger and inferior). The statistical comparison showed a statistically significant difference (*p* < 0.05) in all mean values for DSC, HD95, and MSD, establishing inferior model performance when trained on mixed‐guideline data.

### 3.2 Model performance for delineation guideline classification

**FIGURE 3:**
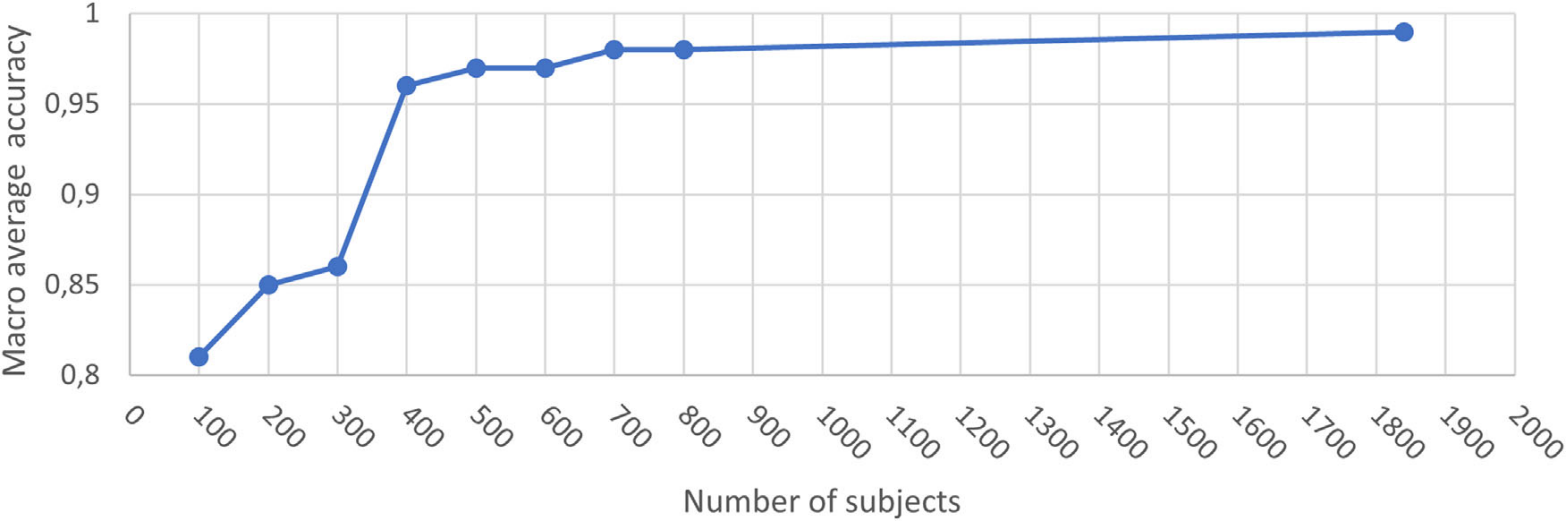
Macro average classification accuracy on validation data in one cross validation fold as a function of number of subjects in the pelvis dataset. A large and steep increase in the classification performance can be seen when number of subjects were increased from 300 to 400.

Model training for each cross‐validation fold using the bowel dataset took about 1 day and 10 h and reached a 98% macro average classification accuracy on the validation data (average of the unweighted mean per label). Corresponding training time for the pelvis dataset was 2.5–5 days per fold (with two GPUs) reaching a validation accuracy of 98%−99% (min‐max). No overfitting during model training was observed for any of the experiments as no cross‐validation fold deviated more than 3% units in the macro average classification accuracy for the validation data compared to the training data. The investigation of the classification performance in one cross validation fold as a function of number of subjects in the pelvis dataset demonstrated that it took more than 300 patients to achieve satisfying classification performance, Figure 3.

The model inference was successful on all test datasets after preprocessing. During preprocessing, performed prior to inference, some structures were ignored due to the limitation in number of 2D slices, and the total number of structures eligible for inference was slightly reduced, see Supplementary tables for number of structures and section 2.3 *Data preprocessing* for conditions. Results, confusion matrixes, and error analysis for the individual test datasets are reported below where RT structure name exclusion was used as default (see section 2.5 *Model inference*).

### 3.3 Bowel test dataset

**FIGURE 4:**
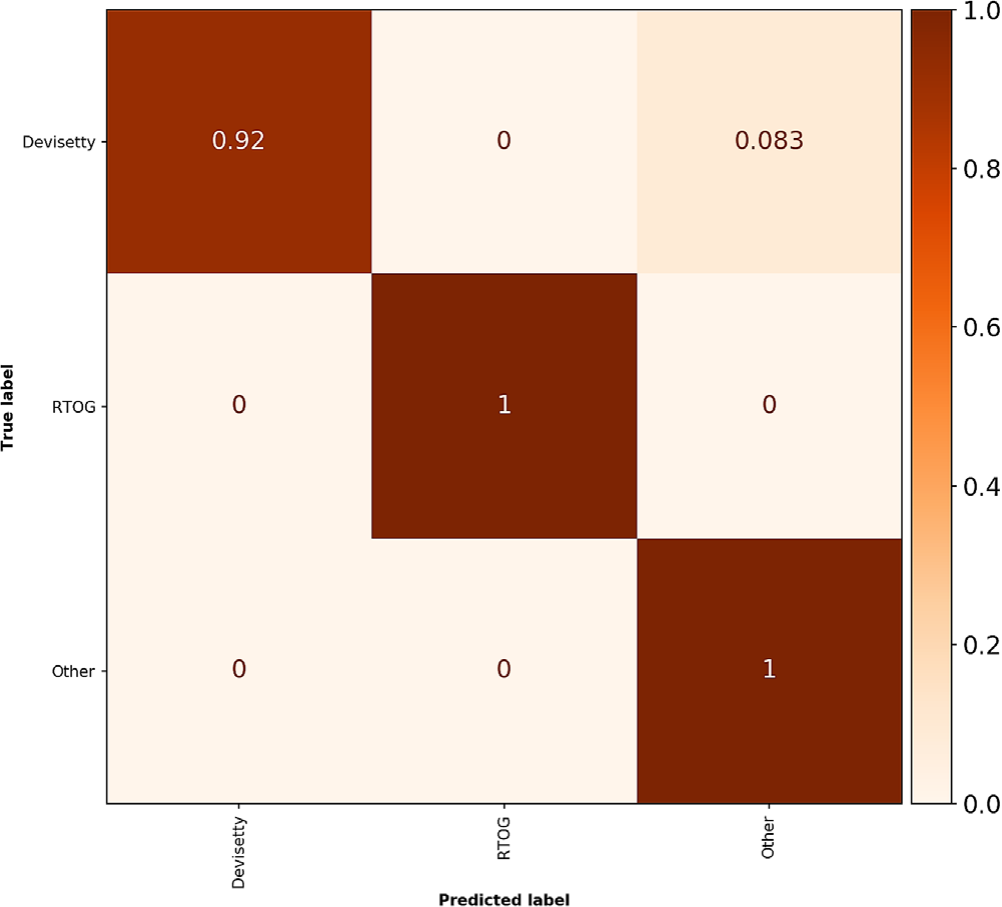
Normalized confusion matrix for the bowel test dataset. A perfect score generates only diagonal values with value 1. Number of objects in each class is given in Table S6.

The bowel test dataset contained 898 structures and model inference could be performed with 19 structures per second. One bowel cavity structure was excluded in the preprocessing step, as it contained more than 96 2D slices, which is currently not supported by the model. Results for the bowel cavity structures are therefore reported for 24 patients. In total, two structures out of the 898 structures were misclassified as belonging to the “Other” class and consisted of two Devisetty bowel structures (see confusion matrix in Figure 4). Unweighted classification accuracy, macro average precision, macro average recall, and macro average F1‐score were 99.8%, 99.9%, 97.2%, and 98.5%, respectively. These values were calculated using RT structure name exclusion. Corresponding values when not using RT structure name exclusion were 99.6%, 97.1%, 97.1%, 97.1%, hence the use of RT structure name exclusion increased all classification metrics. Detailed per‐class evaluation, as well as the evaluation of numbers of structures can be found in Tables S5 and S6. It can be noted that the precision for both bowel structures was 1 when RT structure name exclusion was used (Table S6).

### 3.4 Internal test dataset

**FIGURE 5:**
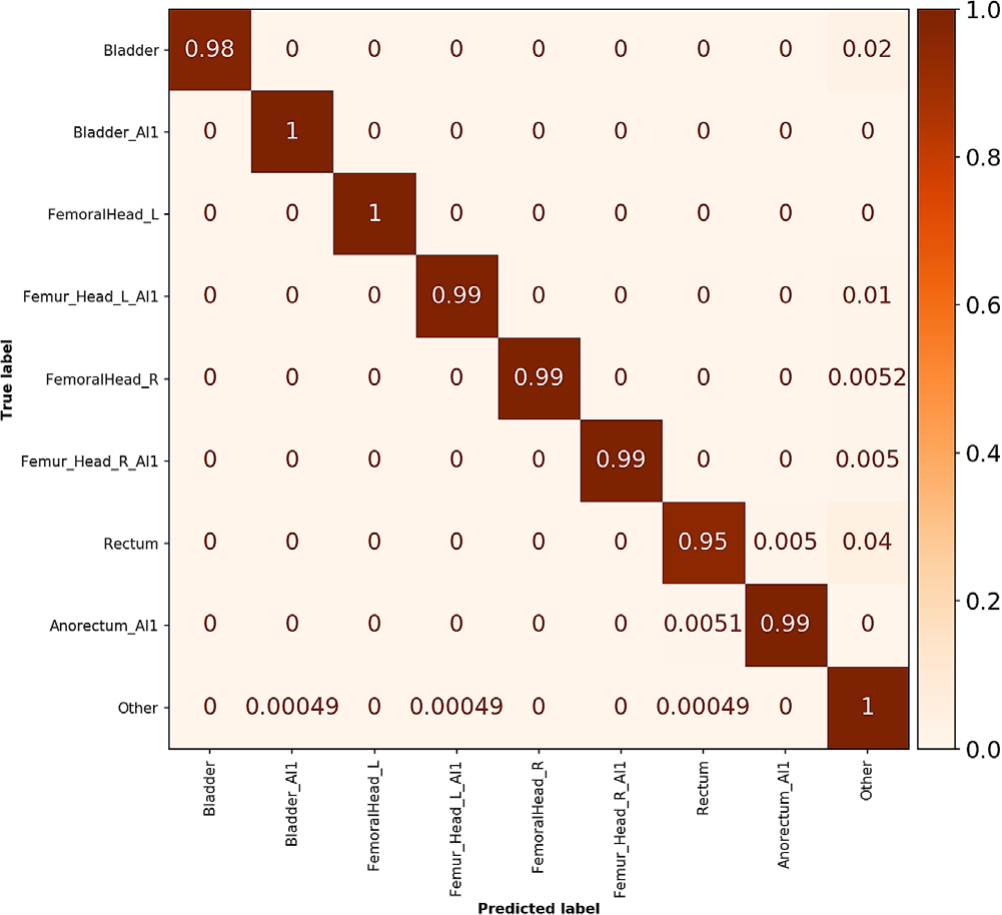
Normalized confusion matrix for the pelvis internal test dataset. A perfect score generates only diagonal values with value 1. Number of objects in each class is given in Table S8.

The internal test dataset contained 200 patients and 3643 structures eligible for inference. The total preprocessing time was measured to be 10 min, and inference could be performed with 17 structures per second. This resulted in a total inference time of 15 min for all five cross validation models. Four DL generated structures (three rectum and one bladder) from the RaySearch male pelvic CT model were incorrectly segmented and positioned far away from the correct organ location. These were after visual inspection redefined in the ground truth as class label “Other.” In total, 21 structures out of the 3643 were misclassified, consisting of 10 rectum, 4 bladder, 4 femoral head, and 3 “Other” structures (see confusion matrix in Figure 5). Unweighted classification accuracy, macro average precision, macro average recall, and macro average F1‐score were 99.4%, 99.6%, 99.0%, and 99.3%, respectively. These values were calculated using RT structure name exclusion. Corresponding values when not using RT structure name exclusion were 99.0%, 98.8%, 98.9%, and 98.8%, hence the use of RT structure name exclusion increased all classification metrics. Detailed evaluation per individual class label and number of structures can be found in Tables S7 and S8.

### 3.5 External test dataset

**FIGURE 6:**
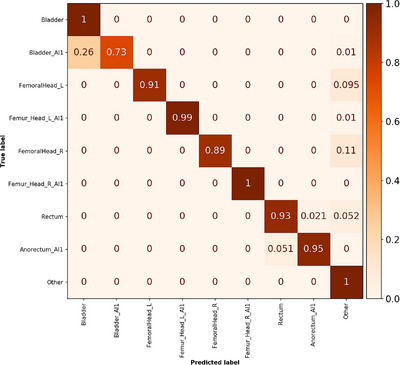
Normalized confusion matrix for the pelvis external test dataset. A perfect score generates only diagonal values with value 1. Number of objects in each class is given in Table S10.

The external test dataset with 99 patients had 1583 structures and inference could be performed with 17 structures per second. One DL generated bladder structure from the RaySearch male pelvic CT model was incorrectly segmented and positioned far away from the true location. This structure was after visual inspection redefined in the ground truth as class label “Other.” In total, 58 structures out of the 1583 was misclassified and consisted of 12 rectum, 26 bladder, and 20 femoral head structures, see confusion matrix in Figure 6. Unweighted classification accuracy, macro average precision, macro average recall, and macro average F1‐score were 96.3%, 96.6%, 93.3%, and 94.6%, respectively. These values were calculated using RT structure name exclusion. Corresponding values when not using RT structure name exclusion were 96.1%, 96.2%, 93.3%, 94.3%, hence the use of RT structure name exclusion increased all classification metrics. As can be seen from the confusion matrix in Figure 6, 26% of the deep learning‐based bladder structure (Bladder_AI1) were misclassified as bladder. This was also the class label with the worst classification performance. In addition, 10% of FemoralHead_R and FemoralHead_L structures were classified as “Other,” and some rectum structures were misclassified as well. Detailed evaluation per individual class label and number of structures can be found in Tables S9 and S10.

## 4 DISCUSSION

In this study, the effects of mixed delineation guideline training data for DL semantic segmentation model training were evaluated and the importance of training data with coherent delineation guidelines for development of DL segmentation applications was demonstrated. To facilitate efficient, precise, and automatic image/structure‐based data extraction, an open‐source deep learning‐based solution for classifying pelvic RT OAR with different delineation guidelines was developed and validated in this study. The proposed solution utilized the relation between the structure delineations and the corresponding CT image volume and was optimized to maximize classification precision. The solution was successfully combined with a label‐name‐dependent exclusion method of RT structures. An image class called “Other” was used for non‐relevant structures (with respect to this application) and validation on both internal and external test dataset showed excellent performance.

Training of semantic segmentation models for bowel using data containing mixed guidelines demonstrated a statistically significant decrease in model performance. Mean DSC was decreased with 3% units, mean HD95 was increased with 5 mm and mean MSD was increased with 1 mm, hence our data indicate that HD95 is a valuable metric for such investigations. The bowel dataset was regarded suitable for such a comparison as the manual delineations for both guidelines were carefully performed by the same oncologist.

Classification models for bowel cavity and pelvic OAR structure delineation guideline were trained using 5‐fold cross validation and were used to classify data with a priority on classification precision. The investigation regarding the dependence on the amount of training data needed demonstrated that around 400 subjects were needed to reach satisfying model performance on validation data, that is, a macro average classification accuracy above 95%.

The classification performance on the bowel test dataset demonstrated excellent performance with a score of 99.8% for unweighted classification accuracy, macro average precision of 99.9%, macro average recall of 97.2%, and macro average F1‐score of 98.5%. The classification precision per each bowel cavity structure class was 1. That means that all objects selected as belonging to a certain bowel cavity delineation guideline class were correct but that the model might have classified some bowel structures as belonging to the “Other” class. This was the case for two bowel cavity structures. This can be regarded as acceptable, as we argue that it is more important to collect the correct data compared to all possible data. This was clearly demonstrated in our segmentation experiment. One patient in the bowel test data was excluded in the preprocessing due to the large number of 2D slices, but the model input size can easily be changed and adapted to a larger 3D matrix size. This would however require retraining of the model. As an alternative, the structure could be downscaled, to fit the input constraints of the network architecture. This was however not investigated for the slice direction in our study.

The pelvis internal test dataset with 200 patients resulted in classification metrics all equal to or above 99% and the misclassification rate was only 0.6%. This was expected as the training cohort was considered to be large. The pelvis external dataset provided a valuable validation of our method as this test data was acquired on a different CT scanner at a different RT clinic and structures were delineated by a different personnel team. The model achieved an unweighted classification accuracy of 96.3% with a macro average precision of 96.6%. All classification performance metrics were 3%−6% units lower compared to the metrics for the internal test dataset. From Figure 6 and Table S10 it can be noted that the most common misclassification occurs for the bladder AI structure, where 26% was misclassified to the bladder structure. 2% of the rectum structures was misclassified to rectum AI and 5% of the rectum AI structures was misclassified to rectum. Surprisingly, around 10% of both left and right femoral head was misclassified as “Other.” This did however not affect the classification precision, that is, femoral head structures determined to the respective classes were correct.

The results discussed above were generated using the RT structure name exclusion method (see section 2.5 *Model inference* for details). The idea of combining the CNN output with existing label text was identified as a future improvement in previous work^18^ and also recognized as valuable in Syed et al.^22^ The RT structure name exclusion provided 0%−3% units increase for the reported classification metrics for all test datasets and motivates the use of RT structure name exclusion. However, it is important to stress that this relatively small increase also means that almost all the classification performance is originating from the deep‐learning model and not text‐based logic, as intended in our method.

The results from this study demonstrated that the use of training data containing a mixture of delineation guidelines is detrimental for DL semantic segmentation performance, that is, a 3% decrease in mean DSC and a 5 mm increase in mean HD95. With regard to the already high mean DSC scores above 90% using mixed guideline training data the decrease in mean DSC was fairly small in relative terms, but we remind the reader that the HD95 measure might be a more valuable metric compared to DSC for such large organs as bowel cavity.^48^ Further, datasets with multiple guidelines delineated by the same oncologist are sparse and the bowel dataset was chosen primarily to demonstrate the detrimental effect, and not chosen from a clinical priority perspective. The lack of multi‐delineation data for other OAR in our dataset limited our investigation to bowel cavity.

With that in mind, it is however possible to differentiate RT delineation guidelines with the use of deep‐learning classification models as we have demonstrated on both internal and external datasets. This will allow for large scale automated and precise data extraction.

To the best of our knowledge, we are the first to address the classification problem for different RT delineation guidelines with a machine learning approach. Our results in this current work can however, at least to some extent, be compared with previous work which have used machine learning for prostate OAR classification.^22^, ^28^, ^32^, ^33^ In these studies, a classification accuracy of 100% have been reported but the number of classes have been limited and validation on independent test datasets have not been performed.^32^ F1 scores of 95% and 90% were achieved on cleaned and uncleaned prostate datasets,^28^ which was lower than our F1 scores.

A detailed comparison on classification performance in this work compared to previous work^18^ is not suitable as the data and number of classes were different. However, internal datasets for both methods scored a classification accuracy of 99% while previous method had inferior results on external test data.^18^ Several important methodological improvements were made in this work compared to previous, such as the implementation of an “Other” class, also used in Syed et al.,^22^ which provided an efficient way of correctly handling optimization and support RT structures, and structures for which the models had not been trained. Results on an external test dataset was thereby largely improved by decreasing false positive classifications. The concept of using AddMap to provide the CNN with useful and important spatial information, also used in Rozario et al.,^32^ was slightly altered compared to the previous work.^18^ The improved version included only the body structure and structure of interest, and not all available structures. Further, the DL models in this work were 3D‐based, as opposed to 2D‐based,^18^ and utilized several important improvements built upon the original ResNet backbone.^41^

The most surprising result in this study was the misclassification of the Bladder AI structure in the pelvis external dataset where 26% of them was classified as Bladder. There was no such Bladder AI structure misclassification for the pelvis internal test dataset. We hypothesize that the reason for this occurrence might be related to the CT images contained in the pelvis external test dataset, as they were acquired with a different CT acquisition protocol and a different CT scanner. During a visual inspection in the error analysis, it was noted that the CT and the bladder structures in the external dataset was in general noisier than the training data. This could affect the outcome in several ways. Firstly, any CT scan differences compared to the training data might influence the classification output, especially since the difference between Bladder and Bladder AI delineations were minor (Figure 1). Secondly, the generation of the Bladder AI structure from the RaySearch male pelvic CT model might be dependent on such scan differences. The effect from this could probably be mitigated by adding image noise and other image modality correlated augmentations to the CT volume during the training of our model. Further, ground truth AI structures should also be generated from CT volumes with added noise. We argue that this could possibly increase the model generalizability.

Another interesting result was the 10% misclassification of femoral heads to the “Other” class for the left and right femoral heads in the pelvis external dataset. After visual inspection of all the misclassified cases it could be noted that the femoral head structures were not delineated as spherical structures, as in the training data, but rather spherical structures where adaptions to the bone anatomy had been made, that is, indentations in the delineated structure. This demonstrated the model's ability to classify the deviating data to the “Other” class, rather than the two available femoral head classes. Similar situations occurred in the pelvis internal test dataset for four femoral head structures which were misclassified to the “Other” class and a more detailed visual evaluation showed that these patients had hip prothesis. This demonstrated once more the model's ability to correctly assign these structures to the “Other” class as the model was not trained on hip prothesis. Further, this highlights the value of image‐based structure classification as the label in these cases did not convey any information that hip prothesis was present.

In the experiment investigating the dependence on macro average classification accuracy as a function of number of subjects in the pelvis dataset, satisfying results on validation data were first reached when the training data contained more than 400 subjects. The detailed analysis showed that the worst classification precision occurred for Bladder and Bladder AI structures and second worst for the Rectum and Rectum AI structures. This is expected due to the subtle difference in the structure delineation guideline between Bladder and the Bladder AI structures (Figure 1a).

The method presented in this work was dependent on supervised training which required data with predefined ground truth labels. We realize that a training dataset with more than 400 patients might be uncommon in the medical field, but we remind the reader that the generation of AI segmentation structures with a commercial software can often be automated for larger patient cohorts. Further, the model input was based on an image matrix covering the body structure. This means that bladder, rectum, and femoral heads will only constitute a minor part of the total image information. By zooming in on each organ, the effective perceptive field of the CNN would be larger, and we hypothesize that the training data needed to separate RT structures with minor delineation guideline differences would decrease. In our work, we used 1854 subjects to train our pelvis model but like to emphasize that only minor classification performance gains were made compared to 400 subjects (Figure 3).

The method described in this paper and the code used to preprocess and train the models is released as an open‐source repository on https://github.com/jamtheim/DicomRTGuidelineClassifierPublic. Custom models can thereby be trained on any structure desired and be used to automatically select high‐quality data from larger datasets. The concept could potentially also be used as an outlier detection tool during the clinical, manual delineation process, to indicate if a delineated structure deviate from the desired delineation guideline. Together with the AI‐based segmentations, it could lead to an increased structure harmonization within a clinic. Future work could be focused on expanding the method to support MRI images and implementation of robust quality control mechanisms. The use of transfer learning, self‐supervised, and semi‐supervised learning methods could potentially be a way forward to eliminate or mitigate the requirement for a large amount of labeled data.^49^

## 5 CONCLUSION

In this work, the importance of coherent RT delineation guidelines in training data for DL semantic segmentation was demonstrated. To facilitate automated, accurate, and fast image data extraction for a specific delineation guideline, a deep‐learning image‐based solution was presented. Internal and external non‐curated test datasets for the pelvic anatomy were evaluated on trained models with excellent results. This allows for data extraction from large datasets while avoiding the need for consistent and correct structure labels.

## AUTHOR CONTRIBUTIONS

Michael Lempart: Participated in the data collection, design of the neural network, and the design of the neural network data input. Michael Lempart contributed substantially to result analysis and interpretation. Michael Lempart is the main author of the work. Jonas Scherman: Provided senior clinical expertise regarding the developed methodology and provided intellectual guidance in the result presentation, analysis, and interpretation. Martin P. Nilsson: Provided senior radiation oncology expertise and provided high‐quality structure delineations. Martin P. Nilsson also provided input regarding the developed methodology and intellectual guidance in the result presentation, analysis, and interpretation. Christian Jamtheim Gustafsson: Designed the study, led, and participated in the data collection, and execution of the study. Christian Jamtheim Gustafsson wrote the code for the method pipeline and carried out the analysis of the data. All authors revised and approved the final manuscript prior to submission.

## CONFLICT OF INTEREST STATEMENT

The authors declare no conflicts of interest.

## Supporting information

Supporting Information
