# Supplementary material for: Deep learning‐based classification of organs at risk and delineation guideline in pelvic cancer radiation therapy
Source: J Appl Clin Med Phys. 2023 May 12;24(9):e14022. doi: 10.1002/acm2.14022 (PMC10476996; doi:10.1002/acm2.14022)
Supplement: Supplementary file 1 — Supporting Information [file ACM2-24-e14022-s001.docx]

# Supplementary material

## Definition of classification metrics

The classification metrics precision, recall, F1 score and accuracy are used in this paper and are hereby defined and explained. The metrics are calculated from true positive (TP), false positive (FP), true negative (TN) and false negative (FN).

Precision: Calculated as TP/(TP+FP). Answers the question what proportion of total positive identifications was actually correct.

Recall (Sensitivity): Calculated as TP/(TP + FN). Answers the question what proportion of total actual positives was correctly identified.

F1 score: Calculated as 2 x Recall x Precision / (Recall + Precision). A weighted average of Precision and Recall.

Accuracy: Calculated as (TP+TN)/(TP+TN+FP+FN). Answers the question what the proportion of correct predations was compared to the total number of predictions.

**Table S1.** ModelDev trained on Devisetty bowel data and applied to test data (n=15) with the same delineation guideline.

| Subject | DSC | HD95 [mm] | MSD [mm] |
| --- | --- | --- | --- |
| 1 | 0.96 | 3.00 | 0.70 |
| 2 | 0.96 | 3.00 | 0.66 |
| 3 | 0.93 | 9.15 | 1.72 |
| 4 | 0.94 | 5.73 | 1.09 |
| 5 | 0.94 | 6.12 | 1.50 |
| 6 | 0.94 | 4.03 | 0.92 |
| 7 | 0.95 | 3.52 | 1.04 |
| 8 | 0.92 | 6.39 | 1.49 |
| 9 | 0.95 | 4.14 | 1.06 |
| 10 | 0.95 | 4.63 | 1.05 |
| 11 | 0.95 | 4.19 | 0.98 |
| 12 | 0.94 | 3.09 | 0.74 |
| 13 | 0.95 | 4.19 | 1.00 |
| 14 | 0.96 | 3.00 | 0.79 |
| 15 | 0.96 | 3.15 | 0.83 |
| Mean | 0.95 | 4.49 | 1.04 |
| SD | 0.01 | 1.72 | 0.31 |

**Table S2.** ModelRTOG trained on RTOG bowel data and applied to test data (n=15) with the same delineation guideline.

| Subject | DSC | HD95 [mm] | MSD [mm] |
| --- | --- | --- | --- |
| 1 | 0.93 | 4.63 | 1.13 |
| 2 | 0.95 | 3.58 | 0.86 |
| 3 | 0.94 | 7.98 | 1.47 |
| 4 | 0.93 | 7.47 | 1.48 |
| 5 | 0.95 | 4.88 | 1.28 |
| 6 | 0.94 | 4.31 | 1.23 |
| 7 | 0.94 | 4.88 | 1.11 |
| 8 | 0.95 | 4.03 | 1.02 |
| 9 | 0.94 | 7.21 | 1.42 |
| 10 | 0.94 | 5.81 | 1.30 |
| 11 | 0.95 | 4.63 | 1.04 |
| 12 | 0.92 | 5.73 | 1.23 |
| 13 | 0.96 | 3.52 | 0.95 |
| 14 | 0.95 | 4.31 | 0.96 |
| 15 | 0.95 | 5.30 | 1.11 |
| Mean | 0.94 | 5.22 | 1.17 |
| SD | 0.01 | 1.38 | 0.19 |

**Table S3.** ModelMix trained on mixed data (50%/50%) and applied to test data (n=15) with the Devisetty delineation guideline.

| Subject | DSC | HD95 [mm] | MSD [mm] |
| --- | --- | --- | --- |
| 1 | 0.92 | 9.62 | 2.34 |
| 2 | 0.94 | 6.00 | 1.29 |
| 3 | 0.93 | 9.21 | 1.83 |
| 4 | 0.91 | 8.37 | 1.85 |
| 5 | 0.94 | 5.09 | 1.40 |
| 6 | 0.90 | 9.21 | 1.92 |
| 7 | 0.92 | 6.31 | 1.64 |
| 8 | 0.92 | 6.65 | 1.59 |
| 9 | 0.84 | 24.63 | 4.69 |
| 10 | 0.91 | 10.64 | 2.55 |
| 11 | 0.93 | 7.16 | 1.74 |
| 12 | 0.86 | 12.65 | 2.45 |
| 13 | 0.92 | 8.73 | 1.86 |
| 14 | 0.95 | 4.88 | 1.27 |
| 15 | 0.95 | 4.98 | 1.21 |
| Mean | 0.92 | 8.94 | 1.97 |
| SD | 0.03 | 4.89 | 0.86 |

**Table S4.** ModelMix trained on mixed data (50%/50%) and applied to test data (n=15) with the RTOG delineation guideline.

| Subject | DSC | HD95 [mm] | MSD [mm] |
| --- | --- | --- | --- |
| 1 | 0.88 | 15.50 | 2.68 |
| 2 | 0.95 | 4.93 | 1.37 |
| 3 | 0.91 | 14.22 | 2.44 |
| 4 | 0.84 | 15.41 | 3.77 |
| 5 | 0.94 | 5.82 | 1.63 |
| 6 | 0.94 | 4.88 | 1.36 |
| 7 | 0.90 | 9.10 | 2.17 |
| 8 | 0.93 | 4.98 | 1.26 |
| 9 | 0.93 | 7.16 | 1.60 |
| 10 | 0.93 | 6.68 | 1.45 |
| 11 | 0.95 | 5.73 | 1.18 |
| 12 | 0.87 | 12.00 | 2.13 |
| 13 | 0.89 | 12.46 | 2.73 |
| 14 | 0.90 | 17.23 | 3.01 |
| 15 | 0.89 | 16.30 | 3.03 |
| Mean | 0.91 | 10.16 | 2.12 |
| SD | 0.03 | 4.72 | 0.80 |

**Table S5.** Classification performance per class label together with macro and weighted average for the bowel test data without “RT structure name exclusion” (25 subjects).

|  | Precision | Recall | F1-score | Support |
| --- | --- | --- | --- | --- |
| Devisetty | 0.9167 | 0.9167 | 0.9167 | 24 |
| RTOG | 1 | 1 | 1 | 24 |
| Other | 0.9976 | 0.9976 | 0.9976 | 850 |
| macro avg | 0.9714 | 0.9714 | 0.9714 | 898 |
| weighted avg | 0.9955 | 0.9955 | 0.9955 | 898 |

**Table S6.** Classification performance per class label together with macro and weighted average for the bowel test data with “RT structure name exclusion” (25 subjects).

|  | Precision | Recall | F1-score | Support |
| --- | --- | --- | --- | --- |
| Devisetty | 1 | 0.9167 | 0.9565 | 24 |
| RTOG | 1 | 1 | 1 | 24 |
| Other | 0.9977 | 1 | 0.9988 | 850 |
| macro avg | 0.9992 | 0.9722 | 0.9851 | 898 |
| weighted avg | 0.9978 | 0.9978 | 0.9977 | 898 |

**Table S7.** Classification performance per class label together with macro and weighted average for the pelvis internal test data without “RT structure name exclusion” (200 subjects).

|  | Precision | Recall | F1-score | Support |
| --- | --- | --- | --- | --- |
| Bladder | 0.9703 | 0.98 | 0.9751 | 200 |
| Bladder_AI1 | 0.995 | 1 | 0.9975 | 199 |
| FemoralHead_L | 0.9949 | 1 | 0.9974 | 195 |
| Femur_Head_L_AI1 | 0.995 | 0.99 | 0.9925 | 200 |
| FemoralHead_R | 0.9897 | 0.9948 | 0.9923 | 194 |
| Femur_Head_R_AI1 | 1 | 0.995 | 0.9975 | 200 |
| Rectum | 0.955 | 0.955 | 0.955 | 200 |
| Anorectum_AI1 | 0.9949 | 0.9949 | 0.9949 | 197 |
| Other | 0.9922 | 0.9908 | 0.9915 | 2058 |
| macro avg | 0.9875 | 0.9889 | 0.9882 | 3643 |
| weighted avg | 0.9899 | 0.9898 | 0.9898 | 3643 |

**Table S8.** Classification performance per class label together with macro and weighted average for the pelvis internal test data with “RT structure name exclusion” (200 subjects).

|  | Precision | Recall | F1-score | Support |
| --- | --- | --- | --- | --- |
| Bladder | 1 | 0.98 | 0.9899 | 200 |
| Bladder_AI1 | 0.995 | 1 | 0.9975 | 199 |
| FemoralHead_L | 1 | 1 | 1 | 195 |
| Femur_Head_L_AI1 | 0.995 | 0.99 | 0.9925 | 200 |
| FemoralHead_R | 1 | 0.9948 | 0.9974 | 194 |
| Femur_Head_R_AI1 | 1 | 0.995 | 0.9975 | 200 |
| Rectum | 0.9896 | 0.955 | 0.972 | 200 |
| Anorectum_AI1 | 0.9949 | 0.9949 | 0.9949 | 197 |
| Other | 0.9923 | 0.9985 | 0.9954 | 2058 |
| macro avg | 0.9963 | 0.9898 | 0.993 | 3643 |
| weighted avg | 0.9942 | 0.9942 | 0.9942 | 3643 |

**Table S9.** Classification performance per class label together with macro and weighted average for pelvis external test data without “RT structure name exclusion” (99 subjects).

|  | Precision | Recall | F1-score | Support |
| --- | --- | --- | --- | --- |
| Bladder | 0.7984 | 1 | 0.8879 | 99 |
| Bladder_AI1 | 1 | 0.7347 | 0.8471 | 98 |
| FemoralHead_L | 1 | 0.9053 | 0.9503 | 95 |
| Femur_Head_L_AI1 | 1 | 0.9899 | 0.9949 | 99 |
| FemoralHead_R | 1 | 0.8947 | 0.9444 | 95 |
| Femur_Head_R_AI1 | 1 | 1 | 1 | 99 |
| Rectum | 0.9091 | 0.9278 | 0.9184 | 97 |
| Anorectum_AI1 | 0.9792 | 0.9495 | 0.9641 | 99 |
| Other | 0.9684 | 0.995 | 0.9815 | 802 |
| macro avg | 0.9617 | 0.933 | 0.9432 | 1583 |
| weighted avg | 0.9645 | 0.9608 | 0.9603 | 1583 |

**Table S10.** Classification performance per class label together with macro and weighted average for pelvis external test data with “RT structure name exclusion” (99 subjects).

|  | Precision | Recall | F1-score | Support |
| --- | --- | --- | --- | --- |
| Bladder | 0.7984 | 1 | 0.8879 | 99 |
| Bladder_AI1 | 1 | 0.7347 | 0.8471 | 98 |
| FemoralHead_L | 1 | 0.9053 | 0.9503 | 95 |
| Femur_Head_L_AI1 | 1 | 0.9899 | 0.9949 | 99 |
| FemoralHead_R | 1 | 0.8947 | 0.9444 | 95 |
| Femur_Head_R_AI1 | 1 | 1 | 1 | 99 |
| Rectum | 0.9474 | 0.9278 | 0.9375 | 97 |
| Anorectum_AI1 | 0.9792 | 0.9495 | 0.9641 | 99 |
| Other | 0.9686 | 1 | 0.984 | 802 |
| macro avg | 0.9659 | 0.9335 | 0.9456 | 1583 |
| weighted avg | 0.9670 | 0.9634 | 0.9627 | 1583 |
